# A scoping review on the associations between early childhood caries and sustainable cities and communities using the sustainable development goal 11 framework

**DOI:** 10.1186/s12903-024-04521-1

**Published:** 2024-06-28

**Authors:** Morẹ́nikẹ ´Oluwátóyìn Foláyan, Elisa Maria Rosa de Barros Coelho, Carlos Alberto Feldens, Balgis Gaffar, Jorma I Virtanen, Arthur Kemoli, Duangporn Duangthip, Ivy Guofang Sun, Ray M. Masumo, Ana Vukovic, Ola B. Al-Batayneh, Tshepiso Mfolo, Robert J Schroth, Maha El Tantawi

**Affiliations:** 1 Early Childhood Caries Advocacy Group, Winnipeg, Canada; 2 Department of Child Dental Health, Obafemi Awolowo University, Ile-Ife, Nigeria; 3 Department of Pediatric Dentistry, Lutheran University of Brazil, Canoas, Brazil; 4 Department of Pediatric Dentistry, Pontifical Catholic University of Rio Grande do Sul, Porto Alegre, Brazil; 5 Department of Preventive Dental Sciences, College of Dentistry, Imam Abdulrahman bin Faisal University, Dammam, Saudi Arabia; 6 Faculty of Medicine, University of Bergen, Bergen, Norway; 7 Department of Paediatric Dentistry and Orthodontics, University of Nairobi, Nairobi, Kenya; 8 Faculty of Dentistry, The University of Hong Kong, Hong Kong SAR, China; 9 Department of Community Health and Nutrition, Tanzania Food and Nutrition Centre, Dar es Salaam, Tanzania; 10 Clinic for Pediatric and Preventive Dentistry, School of Dental Medicine, University of Belgrade, Belgrade, Serbia; 11 Department of Preventive Dentistry, Faculty of Dentistry, Jordan University of Science and Technology, Irbid, Jordan; 12 Department of Preventive and Restorative Dentistry, College of Dental Medicine, University of Sharjah, Sharjah, United Arab Emirates; 13 Department of Community Health, University of Pretoria, Pretoria, South Africa; 14 Dr. Gerald Niznick College of Dentistry, Rady Faculty of Health Sciences, University of Manitoba, Winnipeg, Canada; 15 Department of Pediatric Dentistry and Dental Public Health, Faculty of Dentistry, Alexandria University, Alexandria, Egypt; 16 College of Dentistry, The Ohio State University, Columbus, OH USA

**Keywords:** Sustainable development goal, housing, Urbanization, Waste management, Remoteness, Slums, Natural disasters, Cities, Communities

## Abstract

**Background:**

Early childhood caries (ECC) is a multifactorial disease in which environmental factors could play a role. The purpose of this scoping review was to map the published literature that assessed the association between the Sustainable Development Goal (SDG) 11, which tried to make cities and human settlements safe, inclusive, resilient and sustainable, and ECC.

**Methods:**

This scoping review followed the Preferred Reporting Items for Systematic Reviews and Meta-Analyses Extension for Scoping Reviews (PRISMA-ScR) guidelines. In July 2023, a search was conducted in PubMed, Web of Science, and Scopus using tailored search terms related to housing, urbanization, waste management practices, and ECC. Studies that solely examined ECC prevalence without reference to SDG11 goals were excluded. Of those that met the inclusion criteria, a summary highlighting the countries and regions where the studies were conducted, the study designs employed, and the findings were done. In addition, the studies were also linked to relevant SDG11 targets.

**Results:**

Ten studies met the inclusion criteria with none from the African Region. Six studies assessed the association between housing and ECC, with findings suggesting that children whose parents owned a house had lower ECC prevalence and severity. Other house related parameters explored were size, number of rooms, cost and building materials used. The only study on the relationship between the prevalence of ECC and waste management modalities at the household showed no statistically significant association. Five studies identified a relationship between urbanization and ECC (urbanization, size, and remoteness of the residential) with results suggesting that there was no significant link between ECC and urbanization in high-income countries contrary to observations in low and middle-income countries. No study assessed the relationship between living in slums, natural disasters and ECC. We identified links between ECC and SDG11.1 and SDG 11.3. The analysis of the findings suggests a plausible link between ECC and SDG11C (Supporting least developed countries to build resilient buildings).

**Conclusion:**

There are few studies identifying links between ECC and SDG11, with the findings suggesting the possible differences in the impact of urbanization on ECC by country income-level and home ownership as a protective factor from ECC. Further research is needed to explore measures of sustainable cities and their links with ECC within the context of the SDG11.

**Supplementary Information:**

The online version contains supplementary material available at 10.1186/s12903-024-04521-1.

## Introduction

By 2018, more than half of the global population resided in urban areas, indicating a significant trend towards urbanization [1]. Projections suggest that this number will further escalate to 6.5 billion by the year 2050 [1]. The rise in urbanization is accompanied by an increase in the number of mega-cities. Nine out of ten mega-cities will be in developing countries and about 90% of urban expansion is expected to occur in these nations [1]. Within mega-cities, there are disparities in socio-economic status, with pockets of poverty and deprivation alongside affluent areas often resulting from the growth of urban slums [2]. Presently, the slum population stands at 828 million individuals and is continuously growing [3]. This problem is more pronounced in some countries in Africa like Nigeria, where three out of every five people residing in urban regions reside in slums [4].

These statistics are significant as they highlight the increased risk factor for early childhood caries (ECC) in urban areas. Populations residing in urban slums have challenges accessing adequate oral health services due to structural factors limited availability of dental clinics, shortage of oral health professionals, and long waiting times to access oral health care [5]. In addition, children may individually deal with issues that increase their difficulties accessing nutritious foods, quality oral care products, and regular dental check-ups, all of which further contributes to their high vulnerability to ECC [6, 7]. These factors impede timely preventive and treatment interventions for ECC [5]. On the other hand, children residing in cities may also have a high prevalence of ECC for different reasons [8]. In the cities, there is ready access to unhealthy food options and unhealthy lifestyles, which may contribute to the adoption of unhealthy dietary behaviours such as inadequate fruit and vegetable consumption and increased energy intake [9–12]. For children living in cities and urban slums, the urban environment can increase their exposure to air pollution, which may also contribute to ECC [13]. Therefore, there is a complex interplay of urban-related behavioural, social, economic, and environmental factors that may constitute significant risk factors to the occurrence and progression of ECC.

ECC is defined by any cavitated or non-cavitated lesions in primary teeth in the mouths of children < 72 months of age [14]. The multifaceted risk factors prevalent in urban environments heighten children’s susceptibility to ECC, primarily due to high sugar consumption and exposure to cariogenic diets. Therefore, urban planning must consider the potential impact on children’s oral health, as poor oral health and ECC can impede the achievement of Sustainable Development Goal (SDG) 3. Previous research suggests a correlation between SDG 3 and SDG 11 [15] and a correlation between ECC and SDG 3 [16]. The connection between oral health and SDG 11 remains unclear there is currently no clear evidence on the links between oral health and the SDG 11 [17].

Achieving the SDG 11 may contribute to controlling ECC. The 10 targets of the SDG 11 are focused on improving the quality of life in urban areas, making cities more accessible, safer, and sustainable while mitigating environmental impacts like disasters, ensuring air quality, and waste management. This involves upgrading slums, ensuring basic services and safe transport systems, and creating safe, inclusive, green public spaces. Targets addressing children focus on safe, affordable transport systems and accessible public spaces, benefiting vulnerable groups (SDG 11.2; SDG11.7) [18]. Sustainable cities may also address concerns like air pollution and access to safe waste collection, which is linked with ECC [19, 20], and access to safe waste collection systems, urban green spaces and housing that are also linked to oral health [21–24].

The concept of a smart sustainable city is relatively new with numerous methods and indicators to assess whether the city is smart or sustainable. Its conceptualisation is, however, devoid of health as an indicator though the dimensions and components all contribute to healthy living and wellbeing [25], and possibly oral health. The purpose of this scoping review was to map the evidence in the literature on the association between ECC and targets of the SDG11.

## Methods

We conducted a systematic search to identify literature on the link between sustainable cities and communities and ECC. Our Scoping Review was done in accordance with the Preferred Reporting Items for Systematic Reviews and Meta-Analyses Extension for Scoping Reviews guidelines (PRISMA-ScR) [26].

### Research question

This review was guided by the question: What is the existing evidence on the association between the SDG11 targets (housing, urbanization, waste management practices, natural disasters) and ECC (prevalence and severity)?

### Search strategy

The electronic data searched were PubMed, Web of Science and Scopus, in July 2023. The search terms used are accessible in Appendix 1.

### Selection criteria

Literature obtained through database searches was exported to the reference management software Endnote version 20.6 (Clarivate^™^). Duplicate articles were removed. Title and abstract screening were done independently by two researchers (EMRBC and MOF) using pre-defined inclusion and exclusion eligibility criteria. Full-text reviews of the remaining publications were then done independently by three researchers (EMRBC, CAF and MOF) and reference lists of potentially relevant publications were manually searched. Uncertainty regarding whether publications met the inclusion criteria was resolved through discussion among the three researchers. No authors or institutions were contacted to identify additional sources.

### Inclusion criteria

All epidemiological studies with information on the association between housing, natural disaster, urbanization, and community related factors, and ECC were included. Studies were limited to those that recruited children less than 72 months in keeping with the established case definition for ECC. Publications that included children older than 71 months were included if the findings were appropriately disaggregated by age and enabled the extraction of information and data on children less than 72 months. Publications also had to be peer review articles.

### Exclusion criteria

We excluded studies that did not report ECC as an outcome and those that made no reference to the targets of SDG 11. We also excluded reviews, editorials, case reports and ecological studies. In addition, there were no language restrictions for the search conducted in the databases.

### Data charting

The data extraction process involved gathering specific information from the included publications, such as the first author’s name, publication year, study location, World Health Organization’s (WHO) region where the study was conducted, sample size, age range of the children, study design, and main findings. All relevant information from each publication was compiled and summarized in Table 1 to facilitate a comprehensive analysis. The summarized data was then shared with four experts (MET, BG, JIV and RJS) for their review. Publications were retained only when there was a consensus between the experts and the earlier three reviewers. The final consensus document was also shared with members of the Early Childhood Caries Advocacy Group (www.eccag.org) for validation.

**Table 1:** Information extraction summary

First Author (Year)	Country	WHO region	Sample size/ Age	Study design	ECC prevalence	SDG11 target link	Main findings
Thylstrup (1976) [27]	Denmark	EUR	122 5 years	Cross-sectional	-	11 − 3	No difference in mean dmft among children living in main cities, villages, or small towns.
Tsai (2006) [28]	Taiwan	SEAR	981 1–6 years	Cross-sectional	56%	11 − 3	Children from low urbanization area have the highest caries levels.
Lucas (2011) [29]	Australia	WPR	4606 2–3 years	Cohort	3%	11 − 3	Geographic remoteness of residence is not associated with ECC.
Mantonanaki (2013) [30]	Greece	EUR	524 5 years	Cross-sectional	16.5%	11.1	Children living in rented houses and larger houses have higher dmfs values than those living in private houses
Han (2014) [31]	South Korea	WPR	1214 1–5 years	Cross-sectional	47.5%	11.1	Children living in small-size apartments have higher ECC prevalence than those living in medium or large-size apartments.
Majorana (2014) [32]	Italy	EUR	2395 24–30 months	Cohort	80.8%	11.1	Children living in low-cost houses have higher caries severity levels.
Batliner (2016) [33]	USA	AMR	50 3–4 years	Cohort	76%	11 − 3	The prevalence of untreated ECC of American India children in Santo Domingo Pueblo was similar to previous results reported for the same Indian Health Service Area even though their location is less isolated than many other tribes.
Cabral (2017) [34]	Brazil	AMR	495 4–30 months	Cohort	22.6%	11.1	Children living in a non-masonry house have a higher risk of developing ECC.
Martins-Júnior (2020) [35]	Brazil	AMR	199 5 years	Cross-sectional	43.2%	11.1 11 − 3	ECC prevalence was higher in the smaller municipality, but does not vary according to building material, way waste is managed and the number of rooms in house.
Yazdani (2020) [36]	Iran	EMR	500 4–6 years	Case control	-	11.1	Children from parents who own a house have a lower chance of ECC.

### Data analysis

We performed a descriptive analysis of the publications included in the review, which involved providing detailed information about various aspects of the studies. These descriptions encompassed the countries where the studies were conducted, study design, journal (whether dental or non-dental), prevalence of ECC, and findings regarding the associations between ECC and the indicators of SDG11, which included housing profiles, urbanization, and the impacts of natural disasters. The countries where the studies took place were classified as the Americas region (AMR), Eastern Mediterranean Region (EMR), African region (AFR), European region (EUR), South East Asian region (SEAR), and the Western Pacific region (WPR).

### Role of the funding source

The scoping review was funded by out-of-pocket expenses. This had no role to play in the study design, data collection and analysis, decision to publish, or preparation of the manuscript.

## Results

**Fig. 1:**
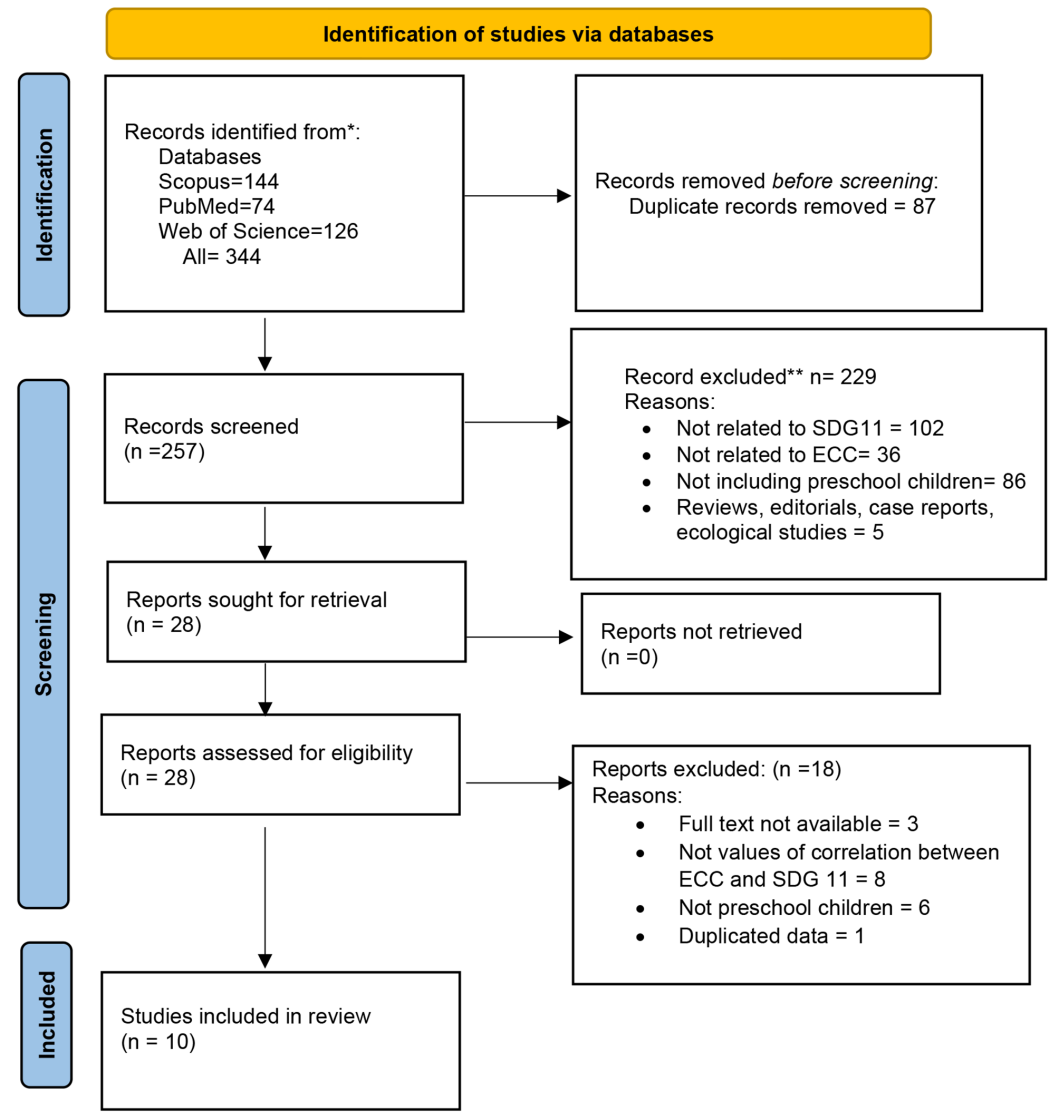
Flow diagram based on the Preferred Reporting Items for Systematic Reviews and Meta-Analyses 2020 flowchart template of the search and selected process

The initial search across three databases yielded 87 potentially relevant publications. After removing duplicates, 65 articles were assessed to determine whether they met the inclusion criteria. Ultimately, only 10 studies were deemed suitable for this scoping review [27–36]. The study selection process is depicted in Fig. 1.

### Characteristics of the study

Table 1 shows that the selected studies were conducted between 1976 and 2020, with only one study conducted before 2000 [27]. Among the 10 studies, five (50.0%) were cross-sectional in design [27, 28, 30, 31, 35], four (40.0%) were cohort studies [29, 32–34], and one (10.0%) was a case-control study [36]. In addition, seven of the 10 studies (70.0%) were published in dental journals [27–31, 35, 36], while three (30.0%) were published in non-dental journals [33–35].

These studies were distributed across the five of the six World Health Organization regions. Three studies (30.0%) were from EUR, with one each from Denmark [27], Greece [30], and Italy [32]. There were two studies (20.0%) from WPR, with one from Australia [29], and one from South Korea [31]; and two studies (20.0%) were from the AMR, with one from the USA [33] and one from Brazil [35]. Additionally, there was one study (10.0%) from the EMR - Iran [36], and one study (10.0%) from SEAR - Taiwan [28], and no study from AFR.

The study populations consisted of children aged between 4 months to 2 years [28, 29, 31, 32, 34] and children aged 3 years to 6 years [28–31, 33–36]. Only One study included children older than 71 months in their sample [36]. Furthermore, two studies had a sample size of less than 350, with one being a cohort study with 50 participants [33] and the other a cross-sectional study with 122 children [27].

### Associations between ECC and SDG 11

Six studies identified an association between housing and ECC [30–32, 34–36] with a study finding suggesting that children whose parents owned a house had lower ECC prevalence [36] and lower ECC severity [30]. However, variables such as living in small apartments [31], low-cost apartments [32], and non-masonry houses [34] were associated with higher ECC prevalence. However, one study found no association between living in masonry houses and ECC prevalence [35] and another showed that children who lived in larger houses had higher ECC severity [30]. There was no difference in ECC prevalence based on the number of rooms in the house [35].

Furthermore, five studies identified a relationship between urbanization and ECC [27–29, 33, 35]. One study found no difference in ECC severity based on the size of the residential area [27], while another showed that children living in smaller municipalities had a higher prevalence of ECC [35]. Two studies indicated that the remoteness of the residential area was not associated with a higher prevalence of ECC [29, 33], while a study showed that children living in low urbanized areas had a higher prevalence of ECC [28].

In addition, among the studies we reviewed, one study investigated the relationship between the prevalence of ECC and waste management practices [35]. The study revealed that there was no significant difference in the prevalence of ECC based on the way waste was managed at the household level, whether it was burned/buried, collected, or left uncollected.

The findings on the links between urbanization and ECC were the most diverse. This variation appears to be associated with the income level of the countries where the research was conducted. Studies from high-income countries did not show a significant link between ECC and urbanization [27, 29, 33]. Conversely, the study from low and middle-income countries indicated that ECC prevalence was higher in low urbanized areas [28]. Based on this analysis, a connection between ECC and SDG11.C is suggested.

There was no study that assessed the relationship between living in slums and ECC, and the relationship between natural disasters such as floods, earthquakes, or hurricanes and ECC.

**Fig. 2:**
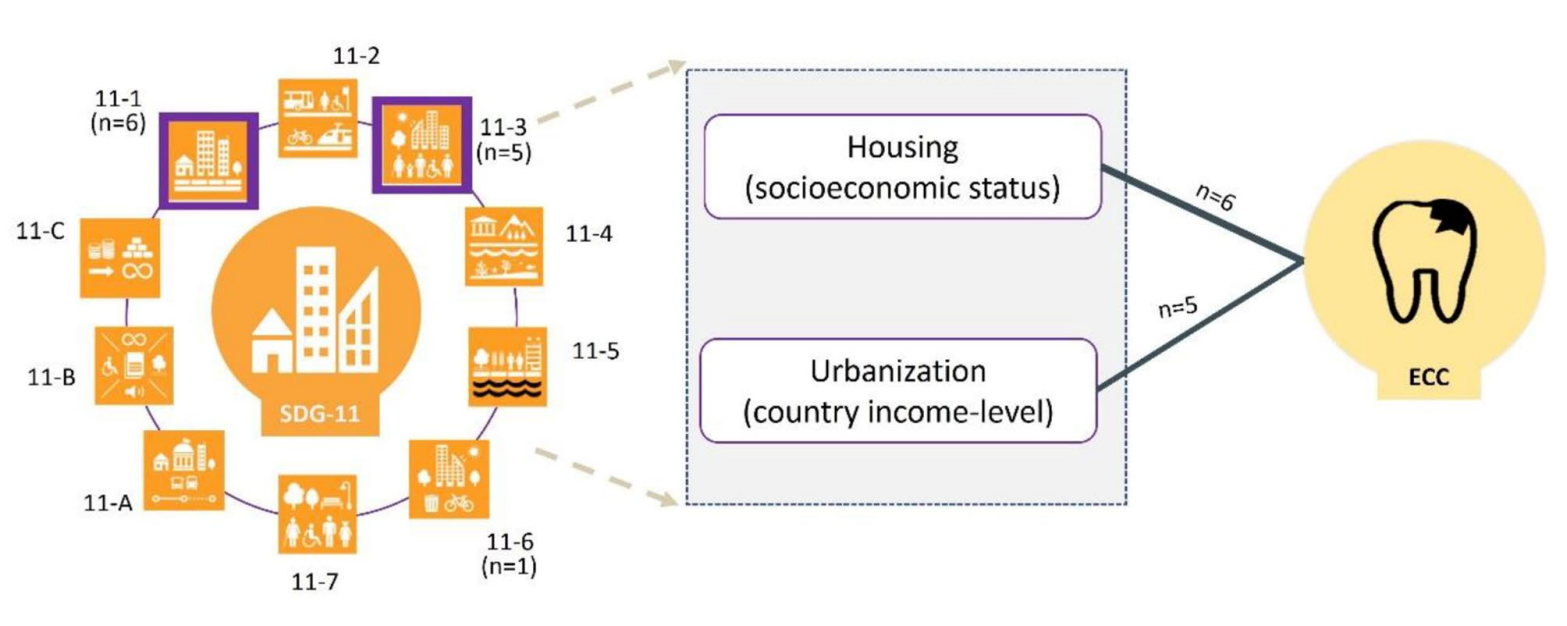
A conceptual framework on the link between ECC and SDG 11 The purple box shows the SDG targets statistically associated with ECC in the current study 11 − 1 safe and affordable housing 11 − 2 affordable and sustainable transport systems 11 − 3 inclusive and sustainable urbanization 11 − 4 protect the world’s cultural and natural heritage 11 − 5 reduce the adverse effects of natural disasters 11 − 6 reduce the environmental impact of cities 11 − 7 provide access to safe and inclusive green and public spaces 11-A strong national and regional development planning 11-B implement polices for inclusion, resource efficiency and disaster risk reduction 11-C support least developed countries in sustainable and resilient building https://knowsdgs.jrc.ec.europa.eu/sdg/11

Figure 2 illustrates our conceptual framework depicting the connection between ECC and SDG11. We identified associations between ECC and SDG11.1 [30–32, 34–36]. The studies that explored the association between SDG11.3 and ECC [27–29, 33, 36] suggest that that the influence of SDG11 on ECC risk could vary based on the income levels of different countries. In lower income countries, urbanization may elevate the risk of ECC, whereas in higher income countries, urbanization might not be linked to the risk of ECC. This finding strengthens the argument of a plausible association between ECC and SDG11.C (which pertains to supporting least developed countries in constructing sustainable and resilient buildings utilizing local materials).

The single study linking SDG 11.6 and ECC [35] found no association between waste management methods and ECC. In addition, we found no study establishing a link between ECC and SDG11.2 (urban transportation access for children), SDG 11.7 (access to safe, inclusive, and accessible, green, and public spaces), SDG11.8 (strengthening national and regional development planning), and SDG11.9 (climate change and disaster management). We did not explore the links between ECC and SDG11.4 (protect and safeguard the world’s cultural and natural heritage) due to the low likelihood of a connection between the two; nor did we explore the link between ECC and SDG 11.5 (economic losses relative to global gross domestic product caused by disasters) because this had been investigated when we explored the link between ECC and SDG8 (decent work and economic growth).

## Discussion

This scoping review was performed to map the existing literature on the links between ECC and SDG11. The study findings revealed that there was a limited number of studies investigating the link between ECC and SDG11. Most of these studies were cross-sectional in design, and the results indicated that owning a house was associated with lower risk of having ECC and lower ECC severity. However, it became evident that other housing-related factors such as apartment size, the number of rooms, and construction materials used may also play a role. The relationship between ECC and urbanization was less distinct, although diverse observations seemed to vary based on the income level of the countries under study. Notably, there were no studies from the African Region. Household waste management did not show a significant association with the prevalence of ECC. This scoping review found studies linking ECC and three SDG 11 targets. There were no accessible studies linking ECC and the other seven SDG 11 targets though there was the plausibility of a link between one of these based on the review of the literature.

The results suggest that having adequate, safe, and affordable housing is associated with lower ECC prevalence and severity. Upgrading slums may also have a positive impact on the risk of ECC, as studies among older children and adolescents have already indicated that living in slums increases the risk for caries [24, 37, 38]. The connections between ECC, housing, and living in urban/rural/remote areas may be related to socioeconomic status, as higher socioeconomic status is associated with improved housing [39], better nutrition and diet [40, 41] and better health [42]. This suggests that the relationship between housing, urbanization and ECC is not causal but rather, housing and urbanization are markers of better socioeconomic status, which is causally linked to ECC risk [43–45]. It is also possible that housing is a marker of better public policies in a neighbourhood, community, or country, and may explain in part, the connection between the SDG 11 and ECC. However, there might be other pathways through which housing and urbanization are linked to the risk and severity of ECC and this needs to be explored by further studies, including doing the difficult task of measuring the direct impact of public policies on oral health.

Some ecological studies have also provided insight into the possible complexity in assessing the links between ECC and SDG11. One of such studies indicated an inverse relationship between urbanization and ECC among European Union member countries [46]. A study conducted on Serbia, one of the European member countries, further corroborated this finding by indicating that residence in parts of a country with lower social and health care expenditures per capita, lower population density, lower local self-government budget and a higher unemployment rate – a profile that may be synonymous with living in a rural or remote area - may increase the risk for both ECC and untreated ECC [47]. It is also possible that urbanization may be linked to higher maternal education, higher income, greater access to information; or it may be a proxy for prompt access to oral health services [48]. The converse may be observed in low middle income countries where a possible pathway linking SDG11 and ECC may be infant feeding practices. Infant feeding practices differ between the urban and rural areas, with infant feeding practices better in the rural area [49]. Urbanization may negatively affect breast feeding practices [50]. These suggest there may be a complex interplay of factors that influence the risk of ECC that housing and urbanization may either moderate or mediate.

In addition, natural disasters may also be linked to the risk of ECC, as they can cause damage to houses including dental facilities and infrastructure such as water and electricity [51]. This increases the risk of poor access to urgently needed preventive dental care and promotes the consumption of cheap foods with high sugar contents, as well as the deterioration of self-care [52]. Previous studies have suggested a causal relationship between economic deterioration and housing damage resulting from natural disasters [51], a decrease in oral health quality of life [53], and the connection between oral disease and insomnia resulting from natural disasters [54]. There were, however, no studies on the link between ECC and natural disasters despite this plausibility. Future disaster management studies should explore the possibilities of this link.

Encouraging further research on the link between ECC and the SDG11 targets, along with exploring plausible connections with other SDG11 targets yet to be studied, holds significant potential for effectively addressing the current high global burden of ECC. Particular attention must be given to the African Region, which currently experiences a substantial burden of untreated ECC [55], high susceptibility to urban migration and the proliferation of slums [56]. Within Africa, attention should be paid to regions in Africa with the highest rate of urban-slum dwellers in the world [57]. The rapid growth of the urban population in Africa, driven by rural-urban migration, is accompanied by poor waste management in these slums [58]. These studies should take into consideration the use of appropriate methodologies including adjustment for socioeconomic status which may diminish the association between the prevalence of caries and urban residency with no impact on the association between caries and semi-urban residency [59]. Therefore, studies exploring the links between ECC and SDG11 may require differentiation between urban, semi-urban, rural, and slum residency. Likewise, access to fluoridated water can attenuate the impact of risk factors on ECC and therefore may also change the relationship between urbanization and ECC [60]. The studies on ECC should profile the risk factors for children 0-2-years old differently from those 3–5 years old [61].

The study had a few limitations. We limited our data extraction to only three databases. As a result, some relevant publications might have been unintentionally excluded. In addition, the scope of our study was restricted to children under six years old, limiting the applicability of our findings to other age cohorts. Despite these limitations, the study underscores plausible connections between ECC and the SDG 11 that warrant empirical exploration in future research especially in Africa.

In conclusion, the findings of this scoping review from the few included studies show there is potential for connections between ECC and SDG11. Firstly, the relationship between housing and the prevalence and severity of ECC indicates a potential mediating role of socioeconomic status. Secondly, studies examining the links between urbanization and ECC were inconclusive, yet hint at possible variations based on country income levels. Thirdly, there were no studies exploring the connections between ECC, living in slums, and natural disasters. Finally, the sole study on waste management practices found no significant association with ECC prevalence. Additionally, the studies were limited to investigating only three of the 10 SDG11 targets. Further research is warranted not only to explore the correlations between ECC and all aspects of SDG11 but also to assess the mediating pathways underlying these connections.

### Electronic supplementary material

Below is the link to the electronic supplementary material.

Supplementary Material 1

## Data Availability

The datasets used and/or analysed for the study are publicly accessible. Data used are summarised in the publication.

## Abbreviations

ECC: Early Childhood Caries
PRISMA-ScR: Preferred Reporting Items for Systematic Reviews and Meta-Analyses Extension for Scoping Reviews guidelines
SDG: Sustainable Development Goal
AMR: Americas region
EMR: Eastern Mediterranean Region
AFR: African region
EUR: European region
SEAR: South East Asian region
WPR: Western Pacific region
