# Supplementary material for: A scoping review on the associations between early childhood caries and sustainable cities and communities using the sustainable development goal 11 framework
Source: BMC Oral Health. 2024 Jun 28;24:751. doi: 10.1186/s12903-024-04521-1 (PMC11214204; doi:10.1186/s12903-024-04521-1)
Supplement: Supplementary file 1 — Supplementary Material 1 [file 12903_2024_4521_MOESM1_ESM.docx]

Appendix 1

Search strategy: Scopus

Bottom of Form

Bottom of Form

| History Count | Search Terms | Results |
| --- | --- | --- |
| 5 | ( TITLE-ABS-KEY ( ( city  OR  cities  OR  {human settlement}  OR  {human settlements}  OR  urban  OR  metropoli*  OR  town*  OR  municipal* )  AND  ( gentrification  OR  congestion  OR  transportation  OR  {public transport}  OR  housing  OR  slum*  OR  {sendai framework}  OR  {Disaster Risk Reduction}  OR  {DRR}  OR  {smart city}  OR  {smart cities}  OR  {resilient building}  OR  {resilient buildings}  OR  {sustainable building}  OR  {sustainable buildings}  OR  {building design}  OR  {buildings design}  OR  urbani?ation  OR  {zero energy building}  OR  {zero energy buildings}  OR  {zero-energy building}  OR  {zero-energy buildings}  OR  {basic service}  OR  {basic services}  OR  {governance}  OR  {citizen participation}  OR  {collaborative planning}  OR  {participatory planning}  OR  {inclusiveness}  OR  {cultural heritage}  OR  {natural heritage}  OR  {UNESCO}  OR  {disaster}  OR  {ecological footprint}  OR  {environmental footprint}  OR  {waste}  OR  {pollution}  OR  {pollutant*}  OR  {waste water}  OR  {recycling}  OR  {circular economy}  OR  {air quality}  OR  {green space}  OR  {green spaces}  OR  {nature inclusive}  OR  {nature inclusive building}  OR  {nature inclusive buildings} ) ) )  AND  ( TITLE-ABS-KEY ( caries )  OR  TITLE-ABS-KEY ( dental  AND caries )  OR  TITLE-ABS-KEY ( dental  AND decay )  OR  TITLE-ABS-KEY ( dental  AND cavities )  OR  TITLE-ABS-KEY ( enamel  AND demineralization )  OR  TITLE-ABS-KEY ( tooth  AND demineralization )  OR  TITLE-ABS-KEY ( tooth  AND cavities ) ) | [144](https://08105cv3w-1105-y-https-www-scopus-com.mplbci.ekb.eg/search/history/results.uri?origin=searchhistory&shid=5) |
| 4 | TITLE-ABS-KEY ( caries )  OR  TITLE-ABS-KEY ( dental  AND caries )  OR  TITLE-ABS-KEY ( dental  AND decay )  OR  TITLE-ABS-KEY ( dental  AND cavities )  OR  TITLE-ABS-KEY ( enamel  AND demineralization )  OR  TITLE-ABS-KEY ( tooth  AND demineralization )  OR  TITLE-ABS-KEY ( tooth  AND cavities ) | [125,206](https://08105cv3w-1105-y-https-www-scopus-com.mplbci.ekb.eg/search/history/results.uri?origin=searchhistory&shid=4) |
| 1 | TITLE-ABS-KEY ( ( city  OR  cities  OR  {human settlement}  OR  {human settlements}  OR  urban  OR  metropoli*  OR  town*  OR  municipal* )  AND  ( gentrification  OR  congestion  OR  transportation  OR  {public transport}  OR  housing  OR  slum*  OR  {sendai framework} OR {Disaster Risk Reduction}  OR  {DRR}  OR  {smart city}  OR  {smart cities}  OR  {resilient building}  OR  {resilient buildings}  OR  {sustainable building}  OR  {sustainable buildings}  OR  {building design}  OR  {buildings design}  OR  urbani?ation  OR  {zero energy building}  OR  {zero energy buildings}  OR  {zero-energy building}  OR  {zero-energy buildings}  OR  {basic service}  OR  {basic services}  OR  {governance}  OR  {citizen participation}  OR  {collaborative planning}  OR  {participatory planning}  OR  {inclusiveness}  OR  {cultural heritage}  OR  {natural heritage} OR {UNESCO} OR {disaster} OR {ecological footprint} OR  {environmental footprint} OR {waste} OR {pollution} OR {pollutant*} OR {waste water}  OR  {recycling}  OR  {circular economy}  OR  {air quality}  OR  {green space}  OR  {green spaces} OR {nature inclusive}  OR  {nature inclusive building}  OR  {nature inclusive buildings} ) ) | [548,414](https://08105cv3w-1105-y-https-www-scopus-com.mplbci.ekb.eg/search/history/results.uri?origin=searchhistory&shid=1) |

Search strategy: WOS

- WOS.SCI: 1900 to 2023
- WOS.AHCI: 1975 to 2023
- WOS.BHCI: 2005 to 2023
- WOS.BSCI: 2005 to 2023
- WOS.ESCI: 2005 to 2023
- WOS.ISTP: 1990 to 2023
- WOS.SSCI: 1900 to 2023
- WOS.ISSHP: 1990 to 2023

| # | Search Query | Results |
| --- | --- | --- |
| 1 | (((((TS=(cities)) OR TS=(human settlements)) OR TS=(urban)) OR TS=(metropoli*)) OR TS=(town*)) OR TS=(municipal*) | 1482219 |
| 2 | (((((((((((((((((((((((((((((((((((((TS=(gentrification)) OR TS=(congestion)) OR TS=(transportation)) OR TS=(public transport)) OR TS=(housing )) OR TS=(slum*)) OR TS=(sendai framework)) OR TS=(Disaster Risk Reduction)) OR TS=(DRR)) OR TS=(smart cities)) OR TS=(resilient buildings)) OR TS=(sustainable buildings)) OR TS=(buildings design)) OR TS=(urbani?ation)) OR TS=(zero energy buildings)) OR TS=(zero-energy buildings)) OR TS=(basic services)) OR TS=(governance)) OR TS=(citizen participation)) OR TS=(collaborative planning)) OR TS=(participatory planning)) OR TS=(inclusiveness)) OR TS=(cultural heritage)) OR TS=(natural heritage)) OR TS=(UNESCO)) OR TS=(disaster)) OR TS=(ecological footprint)) OR TS=(environmental footprint)) OR TS=(waste)) OR TS=(pollution)) OR TS=(pollutant*)) OR TS=(waste water)) OR TS=(recycling)) OR TS=(circular economy)) OR TS=(air quality)) OR TS=(green spaces)) OR TS=(nature inclusive)) OR TS=(nature inclusive buildings) | 2931951 |
| 3 | #2 AND #1 | 440141 |
| 4 | ((((((TS=(caries)) OR TS=(dental caries)) OR TS=(dental decay)) OR TS=(dental cavities)) OR TS=(tooth cavities)) OR TS=(tooth deminerali?ation)) OR TS=(enamel deminerali?ation) | 67415 |
| 5 | #4 AND #3 | 126 |

Search strategy: Pubmed

| # | Query | Results |
| --- | --- | --- |
| 7 | #5 AND #6 | 74 |
| 6 | (((((("Dental Caries"[Mesh]) OR "Tooth Demineralization"[Mesh]) OR (caries[Text Word])) OR (dental decay[Text Word])) OR (dental cavities [Text Word])) OR (tooth cavities[Text Word])) OR (enamel demineralization[Text Word]) | 71,576 |
| 5 | #1 AND #4 | 74,421 |
| 4 | #2 OR #3 | 999,920 |
| 3 | (((((((((((((((((((((((congestion[Text Word]) OR (public transport[Text Word])) OR (slums[Text Word])) OR (sendai framework[Text Word])) OR (Disaster Risk Reduction[Text Word])) OR (DRR[Text Word])) OR (smart cities[Text Word])) OR (resilient buildings[Text Word])) OR (sustainable buildings[Text Word])) OR (buildings design[Text Word])) OR (zero energy buildings[Text Word])) OR (basic services[Text Word])) OR (governance [Text Word])) OR (citizen participation[Text Word])) OR (collaborative planning[Text Word])) OR (participatory planning[Text Word])) OR (inclusiveness[Text Word])) OR (cultural heritage[Text Word])) OR (ecological footprint[Text Word])) OR (environmental footprint[Text Word])) OR (circular economy[Text Word])) OR (air quality[Text Word])) OR (green spaces[Text Word])) OR (nature inclusive[Text Word]) | 128,739 |
| 2 | (((((((((((((("Residential Segregation"[Mesh]) OR "Transportation"[Mesh]) OR "Housing"[Mesh]) OR "Poverty Areas"[Mesh]) OR "Architecture" [Mesh]) OR "Urbanization"[Mesh]) OR "Diversity, Equity, Inclusion" [Mesh]) OR "Cultural Diversity"[Mesh]) OR "UNESCO"[Mesh]) OR "Disasters"[Mesh]) OR "Sewage"[Mesh]) OR ( "Air Pollution"[Mesh] OR "Water Pollution"[Mesh] OR "Environmental Pollution"[Mesh] )) OR "Wastewater"[Mesh]) OR "Recycling"[Mesh]) OR "Parks, Recreational" [Mesh] | 895,307 |
